# Impact of student-run clinic participation on empathy and interprofessional skills development in medical and pharmacy students

**DOI:** 10.1016/j.rcsop.2023.100306

**Published:** 2023-07-10

**Authors:** Karl R. Kodweis, Rachel B. Allen, Emma I. Deschamp, Andrew T. Bihl, David A.M. LeVine, Elizabeth A. Hall

**Affiliations:** a University of Tennessee Health Science Center College of Pharmacy, Memphis, TN, United States of America; b University of Tennessee Health Science Center College of Medicine, Memphis, TN, United States of America

**Keywords:** Student-run clinics, Empathy, Interprofessional, Team-based, Extracurricular

## Abstract

**Background:**

Students participating in student-run clinics (SRCs) have opportunities to develop and practice beneficial skill sets, including empathy and interprofessional collaboration.

**Objectives:**

This study aimed to assess whether participation in an underserved SRC impacts the development of empathy and interprofessional skills in pharmacy and medical students.

**Methods:**

This study assessed empathy and interprofessional skills development through a self-assessment survey. The survey included the Interpersonal Reactivity Index (IRI) to assess empathy, the Attitudes Towards Health Care Teams/Team Skills Scale (ATHCTS/TSS) to assess interprofessional team dynamics, and a free-text response section. Participants were grouped based on whether they participated in the SRC (intervention group) or did not participate in the SRC (control group). A subgroup analysis was performed based on the participants' discipline (medicine vs. pharmacy). To compare differences in IRI, ATHCTS, and TSS scores between study groups, independent samples *t*-tests were performed. A thematic analysis was used for qualitative data.

**Results:**

There were no statistically significant differences between intervention and control groups in IRI, ATHCTS, or TSS scores. Subgroup analyses showed no significant differences in scores of student pharmacists or medical students. For both disciplines, the thematic analysis revealed the most common positive themes identified were “real-world patient interaction and care,” “impact on practice/career development.” Alternatively, it revealed the highest reported negative themes identified as “time management and operational difficulties” and “concerns about the quality of/access to care”.

**Conclusions:**

This study demonstrates that involvement in an SRC neither improves nor hinders a learner's development of empathy and interprofessional team skills. Qualitatively, students reported that participation in an SRC benefited their learning and helped develop their skills, like empathy and team dynamics, in an interprofessional setting. Future research with longitudinal monitoring or alternative assessment tools is recommended.

## 1 Introduction

Student-run clinics (SRC) are practice settings where teams of students from various healthcare-related disciplines work collaboratively to provide primary care services to real-life patients in areas with limited access to healthcare. Several groups have cited benefits from SRCs, including those serving marginalized, underrepresented, and uninsured populations.^1^ In the medical literature, the benefits of participating in an SRC as a student include an increased interest in working with underserved populations, an enhanced understanding of one's role on a healthcare team, and the development of one's empathy.^2^^,^^3^ Huang and colleagues note that students in various healthcare disciplines, including medicine, pharmacy, social work, occupational health, and nursing, found participation in SRCs yields a better understanding of patient-centered care and consideration of diverse professional perspectives compared to their didactic training.^1^ This suggests that participating in SRCs provides healthcare students opportunities to develop and practice skills such as empathy, interprofessional collaboration, and team dynamics.

Empathy is one's ability to experience and understand the emotions of others and plays a vital role in various aspects of healthcare. Two components of empathy include affective and cognitive empathy. Affective empathy refers to one's ability to process an emotional response to another person's situation, while cognitive empathy refers to one's ability to understand another's emotions rationally. Therefore, both are essential skills for healthcare students to develop, as cognitive empathy relies heavily on rationality and procedural thought processes, and affective empathy focuses on feelings and emotions.^4^ Demonstrating empathy, especially in patient care settings, can create more trusting relationships by allowing for more comprehensive information sharing leading to more accurate diagnoses, improved adherence with therapies, and achievement of optimal health outcomes.^5^

Developing interprofessional collaboration skills and team dynamics between future healthcare team members is crucial for ensuring optimal patient care. Literature has shown that interprofessional collaboration improves patient care with diverse patient groups throughout various healthcare settings, including geriatrics, diabetes care, and mental health.^6^, ^7^, ^8^ Furthermore, current medical practice necessitates interprofessional collaboration among healthcare team members to ensure patient safety, improve continuity of care, and standardize the practice of high-quality, patient-centered healthcare.^9^

Despite a recent systematic review by Wilson et al. identifying interprofessional skills and empathy for underserved populations as prominent learning outcomes in student-run clinics, there is a notable gap in the current literature regarding the investigation of the impact of student-run clinic participation on interprofessional team and empathy skill development specifically in pharmacy and medical students.^10^

### 1.1 Objective

This study aimed to assess whether participation in an underserved SRC impacts the development of empathy and interprofessional skills in pharmacy and medical students.

## 2 Methods

### 2.1 Study design and setting

This single-center study was conducted within an SRC at the University of Tennessee Health Science Center (UTHSC). A four-part, self-reported, anonymous survey instrument was developed and sent to eligible participants using Qualtrics (Provo, UT). Before study implementation, the University of Tennessee Health Science Center's (UTHSC) Institutional Review Board approved all study materials, protocols, and background research.

### 2.2 Data collection

All students enrolled in the Doctor of Pharmacy (PharmD) or Doctor of Medicine (MD) programs at UTHSC during the 2021–22 academic year were eligible to participate. The survey was voluntary and anonymous, which meant there were no academic or service requirement consequences for not participating in the study. The pharmacy and medical student leaders at Clinica Esperanza initially dispersed the survey instrument via email and with weekly reminders for its opening duration. All participants were divided into two groups: those who participated in an SRC (intervention group) and those who had not (control group). Those in the intervention group included involvement at Clínica Esperanza or with adjacent experiences defined as “involvement with an extracurricular, student-run practice site.” To determine the inclusion of adjacent experiences in the intervention group, participants included a brief description of their experiences in a free text field, and study investigators were responsible for deciding if those experiences were appropriate. Adjacent experiences were included in the intervention group if the student participated in an SRC, engaged in interprofessional healthcare with other professional students, and the clinic's population was classified as underserved or uninsured.

The SRC studied was Clínica Esperanza (Clinic of Hope), which serves uninsured Hispanic/LatinX patients in Memphis, TN. Every week, student volunteers from the PharmD and MD programs at UTCHS work together to provide healthcare services, often focused on urgent, primary, and ambulatory care-related medical problems, like chronic disease state management. Medical students, under the supervision of an attending physician, serve in a provider role to assess, diagnose, and manage care plans. In conjunction, pharmacy students, under the supervision of an attending pharmacist, work to counsel patients on new medications and newly diagnosed disease states, conduct medication reconciliation, assist in prescription writing, provide recommendations for pharmacotherapy changes, and answer drug-related inquiries from other health disciplines. Furthermore, many students volunteer at Clínica Esperanza regularly and benefit personally and professionally from serving the underserved and uninsured.

### 2.3 Survey instrument

The survey encompassed four components: demographics, the Interpersonal Reactivity Index (IRI) for empathy assessment, the Attitudes Towards Healthcare Teams Scale (ATHCTS) for evaluating interprofessional skill development, and the Team Skills Scale (TSS, Newfoundland, CA). Additionally, a prompt-driven, free-text section was included to examine the impact of participation in an SRC on discipline-specific practice, focusing on the positive and negative effects. The tools to evaluate empathy and interprofessional team skills have previously been validated in pharmacy and medical settings. The IRI has shown reliable internal reliability and has previously been studied in pharmacy students, pharmacists, medical students, medical residents, and physicians.^11^^,^^12^ The ATHCTS has shown reliability and validity in measuring pharmacists, pharmacy students, and graduate students' attitudes towards interprofessional healthcare teams.^13^^,^^14^ When used with the TSS, documented evidence supports the use of ATHCTS and TSS for measuring pharmacy and medical students' perceptions of interprofessional collaboration concerning patient care.^13^^,^^15^^,^^16^

Demographics collected included age, gender, race/ethnicity, professional program enrollment (PharmD or MD), and expected graduation year (e.g., class of 2021).

The IRI used to assess empathy in these cohorts is a survey instrument consisting of 28 items using a 5-point Likert scale (0 = strongly disagree to 4 = strongly agree). The instrument calculates four-different sub-scale scores: perspective taking (IRI-PT), fantasy (IRI-FS), empathic concern (IRI-EC), and personal distress (IRI-PD).^11^ Scores range from 0 to 28 for each subscale, with a total cumulative range of 0 to 112. Higher scores indicate that a participant is more empathetic or has a more significant number of empathetic qualities or traits. Each sub-domain contains seven questions assessing empathy's cognitive and affective components.^11^ IRI-FS, which evaluates one's ability to “place themselves” into another's fictional situation, and IRI-PT, which assesses one's ability to understand another's point of view, assesses cognitive components of empathy. The IRI-EC, which evaluates one's caring feelings for another individual, and the IRI-PD, which considers one's negative feelings upon witnessing adverse effects involving others, assess affective components of empathy.^11^^,^^17^ All participants' scores were collected and analyzed for each sub-domain and in aggregate.

ATHCTS and TSS, often administered in tandem, were used to provide insight into students' self-reported perceptions of interprofessional team dynamics and skills”.^13^^,^18., 19, 20. The ATHCTS is a 20-item instrument with 6-point Likert scale statements (1 = strongly disagree to 6 = strongly agree) to assess a student's perception of healthcare team dynamics. Scores range from 20 to 120, with higher scores indicating more positive attitudes towards interprofessional healthcare teams.^13^^,^^18^ The TSS is a 17-item instrument with 5-point Likert scale statements (1 = poor to 5 = excellent) used to assess students' ability to carry out tasks.^19^ TSS scores range from 17 to 85, with higher scores indicating more positive perceptions of an individual's team-based abilities or competencies.^19^

After completing the IRI, ATHCTS, and TSS assessment tools, those without SRC experiences (control) were finished, and no free text responses were collected. Conversely, a series of free-text questions allowed students with prior SRC participation (intervention group) to describe additional attributes related to their experience beyond what was captured in the quantitative assessment tools. For each of the respective disciplines, the question prompts were, “How has this experience positively affected your pharmacy/medicine practice?” and “How has this experience negatively affected your pharmacy/medicine practice?”

### 2.4 Endpoints

The primary outcome was a comparison of IRI, ATHCTS, and TSS scores between PharmD and MD students with at least one experience participating in an SRC (intervention group) versus those without any experience at an SRC (control group). Secondary outcomes included comparisons of IRI, ATHCTS, and TSS score differences in each program discipline (PharmD and MD).

### 2.5 Data collection

Data collection occurred over a one-month timeframe from December 2021 through January 2022. Following data collection, the survey was closed in February 2022. All data remained within the survey software, Qualtrics (Provo, UT).

### 2.6 Data analysis

Quantitative data were analyzed using IBM SPSS Statistics for Mac (Chicago, IL, v. 27). Data were summarized using descriptive statistics. Between our studied groups, baseline characteristics were analyzed using *t*-tests and Chi-Square analysis to determine the homogeneity of each group. Wilcoxon-Mann Whitney tests were performed to compare differences in IRI, ATHCTS, and TSS scores. An a priori significance level of 0.05 was used. Student free-text responses were analyzed using thematic analysis and reported as frequencies.

Five study investigators conducted a thematic analysis using the Braun and Clarke method on free-text responses from the intervention cohort of pharmacy and medical students.^21^ Four study investigators, representing two student leaders from each discipline, independently reviewed the de-identified free-text responses from both cohorts, focusing on positive and negative aspects. Familiarization with the data involved multiple read-throughs. Each investigator created a set of codes that accurately represented participants' experiences. The codes were then grouped into initial themes and used to determine the frequencies of each theme. These investigators then met to discuss and define the themes, resulting in a comprehensive list. Each investigator independently reviewed the responses based on the group-defined themes and recorded the frequency. Following independent analyses, the group reconvened to discuss their findings. As a group, differences between individual themes identified were reviewed, discussed, and voted upon using a simple majority. Once a consensus was reached, all findings were reviewed by a third-party reviewer not involved in the initial analyses to ensure accuracy.

## 3 Results

The survey was sent to 1289 eligible participants, including MD (*n* = 686) and PharmD (*n* = 603) students. 196 students completed the survey (15.2% overall response rate), with 122 in the PharmD program (20.2% response rate), and 74 in the MD program (10.8% response rate). Demographics revealed a predominantly female and White/Caucasian student population with a mean age of 24.9 years. Racial and ethnicity breakdown between the intervention and control groups showed a significantly higher number of Hispanic/LatinX participants (*p* ≤0.001) in our intervention cohort. A full breakdown of baseline characteristics can be seen in Table 1.

No significant differences in mean IRI, ATHCTS, or TSS scores were noted between the primary groups analyzed, regardless of participation in an SRC (*p*-values ranging from 0.256 to 0.910). A full breakdown can be seen in Table 2.

Regardless of SRC participation, subgroup analysis showed no significant difference in mean IRI, ATCHTS, or TSS scores in the pharmacy (p-values ranging from 0.132 to 0.417) or medicine cohorts (p-values ranging from 0.147 to 0.998). A full breakdown of discipline-specific data for the pharmacy and medicine cohorts can be seen in Table 3, Table 4, respectively.

For the thematic analysis in both cohorts, the positive themes were “real-world patient interaction and care,” “interprofessional experience, teamwork, and collaboration,” “impact on practice/career development,” “helping underserved, non-English-speaking populations,” and “gratitude for involvement and experiences.” In addition, the list of negative themes included: “time management and operational difficulties,” “patient compliance, behavior, or socioeconomic status,” “concerns about the quality of care or access to care,” and “language and cultural barriers.”

Responses on the positive attributes or aspects of participating in an SRC were analyzed for the medical (*n* = 64) and pharmacy students (*n* = 51). The most reported themes identified for both disciplines were “real-world patient interaction and care” and “impact on practice/career development”. To highlight the theme “impact on practice/career development,” one student described SRC participation as “the most impactful part of this experience is [the] real-world practice [of] assessing a patient's chart, conducting a patient interview, creating a patient care plan, and presenting it to both the Supervising Pharmacist and Attending Physician.” Results of the thematic analyses by discipline are in Table 5, Table 6.

“Helping underserved, non-English-speaking populations” was a frequent theme in medicine. One medical student explained that participation within an SRC “…has opened my eyes to the needs not always met by people in our country. I also get exposure to a whole new culture of people -- learning what is important to them.” Another medical student continued with, “…participation helped to “[develop] better empathy… fostered strong relationships with members of the community …[and] the medical community.” While compared to medicine, pharmacy students reported “interprofessional experience, teamwork, and collaboration” at a higher frequency. A pharmacy student said, “It provided my first opportunity to collaborate with students from other disciplines.”

Additionally, responses on the negative aspects of participating were analyzed for the medical (*n* = 21) and pharmacy students (*n* = 23). The most common themes for both disciplines were “time management and operational difficulties” and “concerns about the quality of/access to care.” To highlight “time management and operational difficulties,” a medical student reported, “There are logistical things that could be improved. We would benefit from having an Electronic Medical Record. Paper charts, handwriting, and inconsistent charting can be a hindrance. Additionally, we [could] send referrals much easier.” At the same time, another medical student stated, “I get frustrated when we cannot get things done or when quality care is not being given [due to lack of available resources].” Furthermore, only “[a] small amount of patients [are] seen, the time cost of the language barriers, no-show for follow-up appointments, time not busy or occupied,” as reported by a pharmacy student.

## 4 Discussion

This analysis of IRI, ATHCTS, and TSS scores did not show significant differences between the control and intervention cohorts in aggregate or discipline-specific subgroup analyses. Therefore, there was no statistically significant difference in empathy or interprofessional team skill development between those who participated in an SRC and those who did not.

In a study by Van Hooser and colleagues, pharmacy students who expressed interest in working in underserved areas and ambulatory care had significantly higher IRI scores and a higher level of empathy than their peers not interested in these areas of practice.^12^ Our study did not replicate this previous finding as there was no statistical difference in the level of empathy between students who participated in an SRC and those who did not.^12^^,^^22^

The analysis did, however, show that all student groups analyzed had a high level of empathy and interprofessional team skill development at baseline. At baseline, it has been previously noted that regardless of their academic year, pharmacy students possess an inherent ability to empathize with others.^22^ Furthermore, the lack of significant difference between the intervention and control groups may reflect a student's attributes or values of working with underserved populations, discipline-specific curricular initiatives, similar work or clinical rotation experiences, or additional extracurricular involvement. For example, in the PharmD and MD programs at UTHSC, students must complete service hours each semester as part of their curricula. These requirements are primarily directed at helping the community and underserved populations; thus, these experiences may serve as a confounding variable.

While a significant difference was not detected quantitatively, the thematic analysis revealed that students reported positive attributes to participation in an SRC. The most commonly reported positive features of involvement in an SRC from the medicine cohort was that their experience was humanizing and provided students with an appreciation for patient interactions. One medical student reported that participation at Clínica Esperanza helped to “[develop] better empathy… fostered strong relationships with members of the community …[and] the medical community.” Most positive themes reported among medical and pharmacy students included a real-world application of education and the opportunity to improve patient interactions and develop skills like communication, empathy, and patient interviewing. Notably, both cohorts also reported “interprofessional healthcare team experiences” and “exposure to the social determinants of health” as positive attributes. These findings suggest that participation in an SRC has several positive effects on student empathy and interprofessional team skills.

In a qualitative study, Schutte and colleagues clarified medical students' learning experiences who participated in student-run clinics. They reported three main themes: 1) responsibility for the patient, 2) authenticity of the experience, and 3) interprofessional collaboration with other students and clinical supervisors.^23^ In their study, students found that participation in an SRC was a new, stimulating challenge for students associated with an added level of responsibility. They also noted that SRC participation allowed for an authentic application of their didactic education towards real-world clinical experiences. Finally, their experiences in this setting allowed collaboration with other students and clinical supervisors—fostering an interprofessional, team-based environment. Furthermore, Christopher and colleagues found that participation in an SRC yielded similar educational outcomes compared to traditional simulated experiences commonly utilized in physician, pharmacist, and physician assistant training programs.^24^ Our findings exhibited similar trends in reported themes for both cohorts of students (MD & PharmD). Thus, our study further highlights the benefits of SRC participation and supports previous findings that these real-world, extracurricular experiences produce similar outcomes to clinical simulations and traditional education practices.^24^

To further this, the negative attributes reported of SRC participation indicated authentic problems currently seen in healthcare. Most noted themes addressed the inability to provide comprehensive care due to perceived operational limitations, barriers to patient communication, and interprofessional dissonance between disciplines. One medical student stated, “I get frustrated when we can't get things done or when quality care isn't being given [due to lack of available resources].” And as another pharmacy student stated that only “[a] small amount of patients [are] seen, the time cost of the language barriers, no-show for follow-up appointments, time not busy or occupied.” While these were classified as negative attributes of SRC participation, these represent common barriers to healthcare access seen in the real world. And arguably, exposure to these barriers helps students apply their education and develop their critical thinking skills to provide the best care possible for the patients they see.

This study had several strengths. One strength was the utility of assessment tools, previously validated in medical and pharmacy education, including the IRI, ATHCTS, and TSS. In addition, this was the first study to assess empathy and interprofessional skills development in a population comprising pharmacy and medical students who had participated in an SRC. Furthermore, the study design allowed for both quantitative and qualitative data analysis to provide a more comprehensive picture of the effects of student participation in an SRC.

This study had several limitations. The single-center study design limits its external validity and generalizability to other programs. Regarding the studied population, the sample size was relatively small, contributing to the 15% response rate, and baseline characteristics revealed a lack of diversity between the studied groups. Additionally, for the instruments used, the scores assessed were taken as a “point-in-time” measurement, compared to a longitudinal approach. Using the IRI for assessing empathy development has also shown greater reliability with the general population than in the healthcare setting. Furthermore, student self-reporting and self-evaluation only provide a limited evaluation of empathic ability compared to an external observer's assessment of a student's skill set and may introduce social desirability bias. Finally, we included students with similar or adjacent experiences within the intervention cohort, which could have also contributed to a potential lack of validity and introduced bias.

## 5 Conclusions

In conclusion, this study found that student participation in an SRC neither improves nor hinders a learner's development of empathy and interprofessional team dynamics. Failure to detect a difference between the intervention and control group might result from confounding variables, such as program-specific curricular initiatives, similar work or clinical rotation experiences, or additional extracurricular involvement. Furthermore, quantitative tools that assess empathy and team skills may not provide a comprehensive picture. In our study, the qualitative analysis helped give more insight into the benefits of SRC participation. The thematic analysis revealed that some students who volunteer in an SRC had positive experiences, including their reported development of the same skills investigated herein. Future research exploring a longitudinal monitoring approach of the scores or utilizing an alternative assessment tool, like the Jefferson Scale of Empathy-Health Professions Student, is recommended. A longitudinal analysis may have provided more insight into the development of the skills evaluated over a more extended period. Furthermore, a healthcare-specific assessment tool like the Jefferson Scale of Empathy-Health Professions Student (JSE-HPS) tool may have provided more insight into developing a student's clinical empathy.

## Declaration of Competing Interest

The authors declare that they have no known competing financial interests or personal relationships that could have appeared to influence the work reported in this paper.

## Figures and Tables

**Table 1:** Participant demographics.

	All (*n* = 196)	Intervention (*n* = 82)	Control (*n* = 114)	p-values
Age, mean (SD), *in years*⁎	24.9 (2.8)	26.6 (2.7)	24.4 (2.7)	0.006
Gender, n (%)⁎⁎				
Female	118 (60.2)	51 (62.2)	67 (58.8)	0.606
Male	63 (32.1)	30 (36.6)	33 (29.0)	0.268
Prefer Not to Answer	15 (7.7)	1 (1.2)	14 (12.3)	0.004
Race/Ethnicity, n (%)⁎⁎				
White/Caucasian	138 (70.4)	60 (73.2)	78 (68.4)	0.465
Asian	27 (13.8)	11 (13.4)	16 (14.0)	0.889
Black/African American	18 (9.2)	11 (13.4)	7 (6.1)	0.084
Hispanic/LatinX	11 (5.6)	10 (12.2)	1 (0.9)	<0.001
Prefer not to answer	11 (5.3)	0 (0.0)	11 (9.7)	0.394
Other	3 (1.5)	2 (2.4)	1 (0.9)	0.382
Anticipated Graduation Year, n (%)⁎⁎				
Class of 2022	28 (14.3)	14 (17.1)	14 (12.3)	0.352
Class of 2023	48 (24.5)	27 (32.9)	21 (18.4)	0.013
Class of 2024	64 (32.7)	25 (30.5)	39 (34.2)	0.448
Class of 2025	56 (28.6)	16 (19.5)	40 (35.1)	0.022
Professional Program, n (%)⁎⁎				
Medicine (MD)	74 (37.8)	49 (59.8)	25 (21.9)	<0.001
Pharmacy (PharmD)	122 (62.2)	33 (40.2)	89 (78.1)	<0.001

⁎ T-tests were utilized to determine statistical significance (p > .05).

⁎⁎ Chi-Square tests were utilized to determine statistical significance (p > .05).

**Table 2:** Comparison of empathy (IRI scores) and interprofessional team dynamics (ATHCTS and TSS) for PharmD and MD students participating (intervention) vs. not participating (control) in a student-run clinic.

	All (*n* = 196)	Intervention (*n* = 82)	Control (*n* = 114)	*p*-value
IRI, mean (SD)	71.4 (10.6)	71.3 (9.9)	71.4 (11.1)	0.910
Perspective	19.8 (3.5)	19.7 (3.5)	19.9 (3.4)	0.726
Fantasy	18.8 (4.7)	19.1 (4.7)	18.5 (4.8)	0.380
Empathy	21.4 (3.8)	21.5 (3.5)	21.3 (4.0)	0.672
Personal Distress	11.9 (4.5)	11.4 (4.1)	12.2 (4.8)	0.220
ATHCTS, mean (SD)	87.0 (7.5)	87.2 (5.7)	86.9 (8.6)	0.285
TSS, mean (SD)	60.1 (10.1)	59.1 (8.2)	60.7 (11.2)	0.256

ATHCTS, Attitudes Towards Healthcare Teams; IRI, Interpersonal Reactivity Index; MD, Doctor of Medicine; PharmD, Doctor of Pharmacy; TSS, Team Skills Scale.

**Table 3:** Comparison of empathy (IRI scores) and interprofessional team dynamics (ATHCTS and TSS) for PharmD students participating (intervention) vs. not participating (control) in a student-run clinic.

	All (*n* = 122)	Intervention (*n* = 33)	Control (*n* = 89)	p-value
IRI, mean (SD)	70.2 (10.9)	68.3 (9.1)	70.9 (11.5)	0.198
Perspective	19.6 (3.4)	18.8 (3.6)	19.9 (3.4)	0.148
Fantasy	18.0 (4.6)	17.5 (3.9)	18.2 (4.8)	0.410
Empathy	20.9 (3.9)	20.4 (3.3)	21.1 (4.2)	0.366
Personal Distress	12.2 (4.5)	12.0 (3.3)	12.2 (4.9)	0.753
ATHCTS, mean (SD)	87.4 (7.1)	88.2 (5.5)	87.2 (7.6)	0.417
TSS, mean (SD)	61.6 (10.1)	59.6 (8.2)	62.4 (10.7)	0.132

ATHCTS, Attitudes Towards Healthcare Teams; IRI, Interpersonal Reactivity Index; PharmD, Doctor of Pharmacy; TSS, Team Skills Scale.

**Table 4:** Comparison of empathy (IRI scores) and interprofessional team dynamics (ATHCTS and TSS) for MD students participating (intervention) vs. not participating (control) in a student-run clinic.

	All (*n* = 74)	Intervention (*n* = 49)	Control (*n* = 25)	*p*-value
IRI, mean (SD)	73.2 (9.8)	73.2 (10.1)	73.2 (9.5)	0.998
Perspective	20.2 (3.4)	20.3 (3.3)	19.9 (3.7)	0.648
Fantasy	20.0 (4.8)	20.2 (4.9)	19.6 (4.7)	0.595
Empathy	22.2 (3.4)	22.3 (3.5)	22.1 (3.4)	0.844
Personal Distress	11.5 (4.5)	11.1 (4.6)	12.2 (4.4)	0.304
ATHCTS, mean (SD)	86.4 (8.2)	86.5 (5.9)	86.0 (11.7)	0.848
TSS, mean (SD)	57.4 (9.5)	58.7 (8.3)	55.0 (11.3)	0.147

ATHCTS, Attitudes Towards Healthcare Teams; IRI, Interpersonal Reactivity Index; PharmD, Doctor of Pharmacy; TSS, Team Skills Scale.

**Table 5:** Thematic analysis of medical students' free responses on the effects of participation in an SRC.

Attributes of Participation	Examples
Positive (*n* = 64)	
Real-world patient interaction and care	*“The most impact aspect of this experience was because it was one of my first experiences with patients and it showed me how to speak with them, but also introduced me to a population much different than I had grown up knowing well.”* *“I also have learned so much about how to treat patients and how to develop my bedside manner. It has been my most meaningful medical school experience.*
Interprofessional experience, teamwork, and collaboration	*“I have found the team aspect and the teaching and learning opportunities this has provided to be the most impactful for me.”* *“Finally, working closely with pharmacy students gave me a much better understanding of the breadth of their skills”*
Impact on practice/career development	*“I learned valuable skills while working at Clinica Esperanza, including those related to primary care and determining the best treatment plan for each patient.”* *“It has allowed me to practice with an underserved population, gain valuable clinical skills and experiences, and work with patients to provide care when there is a barrier.“*
Helping underserved, non-English-speaking population	*“It has opened my eyes to the need that is not always being met by people who live in our country. I also get exposure to a whole new culture of people -- learning what is important to them”* *“This experience has helped to open up the opportunity to serve populations in need, and to give me the experience to work with them in a clinical setting. It has built my passion to serve those in underserved areas to a level higher than I thought.”*
Gratitude for involvement, positive experiences	*“School can be incredibly stressful and volunteering at Clinica is a good reminder of what I'm working towards.”* *“It was positive from an educational and social perspective.”*
Negative (*n* = 21)	
Time management, operational difficulties	*“There are logistical things that could be improved. We would benefit from having an Electronic Medical Record. Paper charts, handwriting, and inconsistent charting can be a hindrance. Additionally, we can send referrals much easier.”* *“I get frustrated when we can't get things done or when quality care isn't being given [due to a lack of available resources].”*
Patient compliance, behavior, or socioeconomic status	*“It is disheartening to see how one's socioeconomic status affects access to certain health practices.”* *“Barriers exist that make it difficult for uninsured immigrant patients to access to health system“*
Concerns about the quality of care, access to care	*“It has been frustrating to see patients with complex, dire medical issues be treated primarily by students and receive a couple of minutes of care from an experienced physician”* *“It has jaded me to the unethical and unjust medical system and the ways officials perpetuate the system at the expense of the most vulnerable in our community.”*

**Table 6:** Thematic analysis of pharmacy students' free responses on the attributes of participation in an SRC.

Attributes of Participation	Examples
Positive (*n* = 51)	
Real-world patient interaction and care	*The most impactful part of this experience is getting real-world practice assessing a patient's chart, conducting a patient interview, creating a patient care plan, and presenting it to both the Supervising Pharmacist and Attending Physician.* *Communicating about a care plan for the patient helped me apply my knowledge to the real world.*
Interprofessional experience, teamwork, and collaboration	*It provided my first opportunity to collaborate with students from other disciplines.* *It gave me the opportunity to work with doctors and medical students.*
Impact on practice/career development	*This experience has strengthened my comfort in interacting with physicians and helped me with confidence in my recommendations.* *Really learning the role, we play on the team and how to interact with medical students. I had always wondered how that works and I value that experience before I go to rotations.*
Helping underserved, non-English-speaking population	*I feel more confident in my ability to work with diverse patient populations including patients with barriers to care such as speaking a different language, lack of insurance, and lack of financial resources.* *I gained a different perspective into the importance of providing proper medical care to those who cannot afford health insurance.*
Gratitude for involvement, positive experiences	*The most impactful aspect was how grateful the patients were. They were so happy to receive care when they otherwise would not have been able to.* *During a counseling session with a Spanish-speaking patient, I had to get an interpreter for this conversation, and she challenged me with some hard questions about her medication. This was a special experience because I was able to learn how to counsel patients better, and was touched by how grateful she was to be there.*
Negative (*n* = 23)	
Language and cultural barriers	*It is difficult to engage with others from a different background for many reasons. Cultural and religious barriers concerning vaccines and other interventions must be considered.* *It is sad to see how many people need help and do not get what they need just because of language barriers and cultural barriers.*
Time management, operational difficulties	*What bothered me was the amount of time an appointment took. The patient was there for 4* *h and still wasn't done- we just ran out of time. She also did not get the prescription she needed.* *A small amount of patients [are] seen, the time cost of the language barriers, no-show for follow-up appointments, time not busy or occupied.* *Some of the time I was not busy or occupied, which can feel like a waste of time.*
Patient compliance, behavior, or socioeconomic status	*It is difficult to confirm my patient's medication history when he did not remember the exact dose or how he should be taking them*
Concerns about the quality of care, access to care	*Not being a fluent speaker, has made me want to learn more Spanish and specific medical terms that may be hard to understand in English. Therefore, I can give my patient the proper care they deserve.* *Sometimes, patients who doesn't want to listen to student's recommendations*
